# The impact of ^222^Radon escape on ^210^Pb-based sediment chronology and dating accuracy in lacustrine systems

**DOI:** 10.1038/s41598-026-56349-0

**Published:** 2026-06-10

**Authors:** Robert-Csaba Begy, Anita Bozsik, János Korponai, Enikő Katalin Magyari, Tibor Kovács

**Affiliations:** 1 Faculty of Environmental Science and Engineering, “Babeş-Bolyai” University, Cluj-Napoca, Romania; 2 Interdisciplinary Research Institute on Bio-Nano-Science, Babeş-Bolyai University, Cluj-Napoca, Romania; 3 National University of Public Service, Baja, Hungary; 4 Department of Environmental and Landscape Geography at Eötvös Lóránd University, Budapest, Hungary; 5 Department of Biophysics and Radiation Biology-HUN-REN TKI, Semmelweis University, Budapest, Hungary

**Keywords:** Environmental sciences, Solid Earth sciences

## Abstract

The accuracy of ^210^Pb-based sediment chronology relies on determining atmospheric ^210^Pb inventory by subtracting ^226^Ra-supported ^210^Pb. This standard analytical approach assumes a closed system where the gaseous intermediate, ^222^Rn, remains in secular equilibrium with ^226^Ra. However, in lacustrine systems characterized by slow sedimentation and high porosity, ^222^Rn escape can invalidate this assumption, significantly distorting age models. This study investigated ^222^Rn mobility across three distinct systems: Lake Balaton (Hungary), Lake St. Anna, and Red Lake (Romania). In Balaton, high ^226^Ra (25–55 Bq/kg) and porosity (70–90%) induced significant ^222^Rn loss (3 ± 0.2–37 ± 4 Bq/l) within the upper 30 cm, leading to in situ ^210^Pb deficits (14–56%) and chronological errors reaching 200%. Lake St. Anna, with slow sedimentation and high organic content, showed the highest discrepancies (8–87%) despite lower ^222^Rn levels (0.2 ± 0.01–3 ± 0.2 Bq/l). Conversely, Red Lake exhibited the smallest deviations (1–32%) due to faster accumulation and sandy sediment, notwithstanding measurable ^222^Rn escape (2 ± 0.2–28 ± 4 Bq/l) relative to its ^226^Ra content (35 Bq/kg). The results confirm that these sediments cannot be treated as closed systems regarding radon. Significant gas escape leads to an underestimation of supported ^210^Pb if equilibrium is assumed. We conclude that pore water analysis and subsequent correction for radon diffusion are critical for reliable ^210^Pb chronologies, particularly in slow-accumulating, porous sediments spanning the last 150 years.

**Supplementary Information:**

The online version contains supplementary material available at 10.1038/s41598-026-56349-0.

## Introduction

The ^210^Pb-based geochronological method is widely utilized today, as it spans the specific timescale of intensifying anthropogenic influence on the environment. The past two centuries have been marked by industrialization and the associated rise in carbon dioxide emissions. Investigating the imprints of these processes in natural archive systems, such as lake sediments, peat bogs, or ice cores^1^, is impossible without accurately determining the age of the events recorded within them^2–8^.

The ^210^Pb method is based on a naturally occurring radionuclide within the ^238^U decay series. It originates from the decay of ^226^Ra in soils, which produces the noble gas ^222^Rn. Upon emanation into the atmosphere, radon decays through a sequence of short-lived progeny to form ^210^Pb (T_1/2_ = 22.3 years). Following atmospheric deposition, the specific half-life of ^210^Pb makes it an ideal tracer for dating environmental processes over the past 200 years^9–13^.

In sediments, soils, and peat deposits, the total ^210^Pb activity consists of two distinct components: the unsupported (or excess) fraction derived from atmospheric fallout, and the supported fraction produced by the in-situ decay of ^226^Ra within the mineral matrix. Age determination relies on the unsupported component. Despite being an absolute dating method, ^210^Pb geochronology is subject to several significant uncertainties that can compromise its precision^14^. The measurement of ^210^Pb is complex: gamma spectrometry, commonly used for this purpose, is the least precise due to the low energy and low emission probability of the ^210^Pb gamma transition; however, well-type detectors offer a highly efficient alternative for this measurement. Liquid scintillation counting (LSC) based on the detection of ^210^Bi suffers from quenching effects, while the analytically most precise approach, measuring ^210^Po as a proxy for ^210^Pb, faces challenges arising from the differing chemical behavior of lead and polonium.

Furthermore, the mathematical models used for ^210^Pb dating rely on multiple assumptions, introducing additional uncertainties into the age–depth models^15^. To address these limitations, the application of artificial isotopes as secondary chronostratigraphic indicators has become essential. During model development, it was generally accepted that unsupported ^210^Pb can be obtained by subtracting the ^226^Ra-derived supported ^210^Pb component from the total ^210^Pb concentration, assuming equilibrium within the system^10,12^. This assumption, however, remains valid only if the system is closed to radon-222 (^222^Rn) loss. When ^222^Rn escapes from the system, the equilibrium between ^226^Ra and ^210^Pb is disrupted, as the decay of ^210^Pb is no longer balanced by its production within the decay series. This issue is particularly significant in lacustrine sedimentary environments (whereas in peatlands, the ^226^Ra concentration is typically insufficient to produce measurable supported ^210^Pb fraction). Numerous studies have demonstrated that sedimentary systems do not function as a closed systems with respect to ^222^Rn gas^16–18^.

In several studies, the supported ^210^Pb concentration has been estimated from the deepest sediment layer where the total ^210^Pb concentration becomes nearly constant^11,19–21^. This approach is problematic, as deeper layers may exhibit negligible or significantly reduced radon flux compared to the overlying strata, leading to uncertainty in the estimation of supported ^210^Pb. This uncertainty, combined with the aforementioned measurement and modelling inaccuracies, significantly affects the reliability of ^210^Pb-derived chronologies.

The present study addresses these limitations by determining the sediment–water radon profiles in three lakes with contrasting origins and sedimentological characteristics. Sampling was carried out in Lake Balaton (Hungary), as well as Red Lake and Lake St. Anna (Romania). Based on the obtained radon profiles, we quantify the supported ^210^Pb fraction and evaluate the extent of the uncertainty introduced into the age model when supported ^210^Pb is calculated using either the measured ^226^Ra or from the minimum ^210^Pb concentration observed in the deep sediment layers. Given the strong correlation between ^222^Rn and sediment porosity, we propose a mathematical approach for estimating supported ^210^Pb, thereby establishing a more accurate and reliable age–depth model for sedimentary sequences.

## Results and discussions

**Fig. 1:**
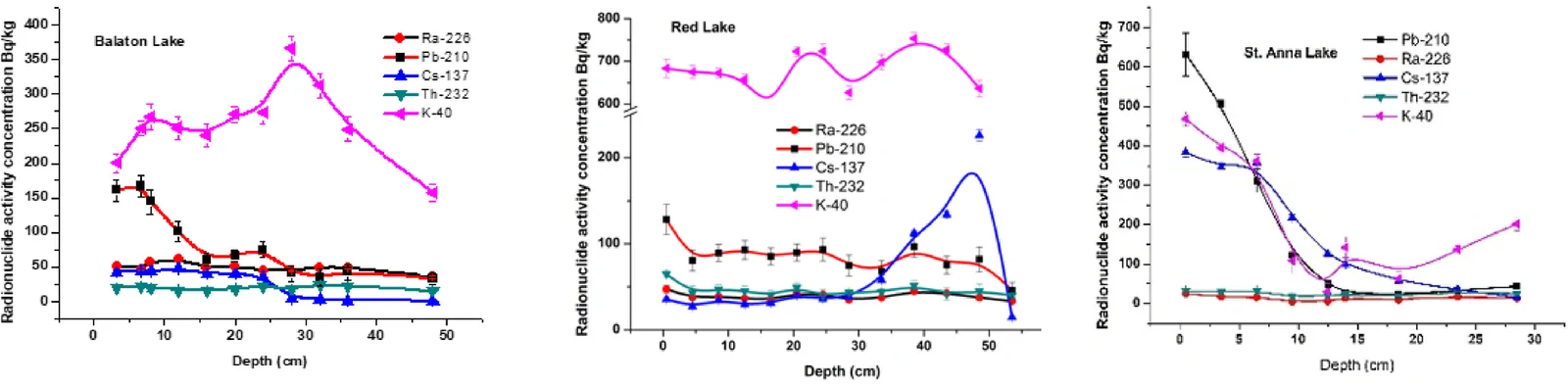
Distribution of radionuclide activity concentrations in the investigated sediment cores: (**a**) Lake Balaton, (**b**) Red Lake, (**c**) Lake Saint Anna.

The results obtained from the radiometric measurements are presented in Fig. 1. A notably high activity concentration of ^210^Pb is observed in the case of Lake Saint Anna. Based on previous investigations^22^, this can be explained by the low sedimentation rate, which results in a large proportion of atmospheric ^210^Pb being retained in the upper sediment layers. The sediment is rich in organic matter, providing an enhanced binding capacity for lead. Notably, alpha spectrometric measurements of the ^210^Po isotope were also performed on the Lake Saint Anna sediment core, confirming the data obtained by gamma spectrometry.

Regarding ¹³⁷Cs, two distinct peaks are identifiable within the sediment cores, providing an alternative dating method with two precisely defined temporal horizons, except in the case of Red Lake.The shallow depth of this peak in the Lake Saint Anna core further supports the interpretation, as it occurs at a depth of only 7 cm below the sediment interface. In contrast, in the Red Lake sediment core, the peak is located at 48 cm depth; here, the peak originating from the 1963 weapon tests does not appear. It is important to note regarding Red Lake that the ²¹⁰Pb concentration and the position of the ¹³⁷Cs peak clearly indicate that the core did not reach the dating horizon—that is, the point where atmospheric lead concentration is no longer detectable. In the case of Lake Balaton, both ¹³⁷Cs peaks are visible (Fig. 4); the peak from the 1986 Chernobyl reactor accident appears at a depth of 11 cm, whereas the peak associated with the 1963 nuclear test ban treaty occurs at 21 cm.

Variations in ^226^Ra and ^232^Th activity concentrations follow similar trends across all investigated lakes, suggesting a common source. Conversely, ^40^K activities vary distinctly within each sediment core, though values remain within the same order of magnitude. These variations are likely related to the proportion of inorganic and organic components, and—where correlated with other isotopes—may also reflect fluctuations in sedimentation rate. A comprehensive discussion of this aspect is beyond the scope of the present study.

**Fig. 2:**
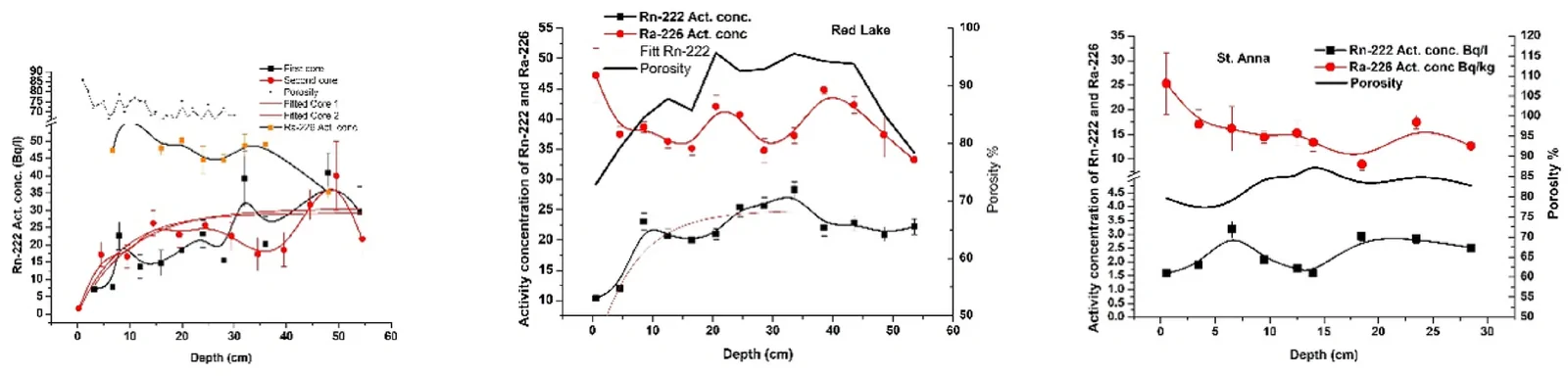
Porosity, ^226^Ra and ^222^Rn concentration in pore water in the investigated lakes.

Figure 2 presents the depth distribution of ^222^Rn concentration in the pore water. The figures include porosity data as well as ^226^Ra concentrations.

From Fig. 2, it is evident that in the upper layers—where ^210^Pb dating is applied to investigate approximately the last 200 years—the expected equilibrium between ^226^Ra and its decay product ^222^Rn is not maintained. Consequently, the initial ^226^Ra–^210^Pb equilibrium within the sediment layer becomes progressively disturbed, resulting in a decrease in in situ ^210^Pb concentration.

As mentioned above, precise knowledge of the in situ ^210^Pb concentration is essential for the construction of a reliable age model, since the measured total ^210^Pb activity must be corrected by this value to obtain the atmospheric (unsupported) ^210^Pb component, which forms the basis of the chronology. The difference between the ^226^Ra concentration of the sediment layers and the ^222^Rn concentration measured in pore water determines the magnitude of the previously defined *k* factor.

**Fig. 3:**
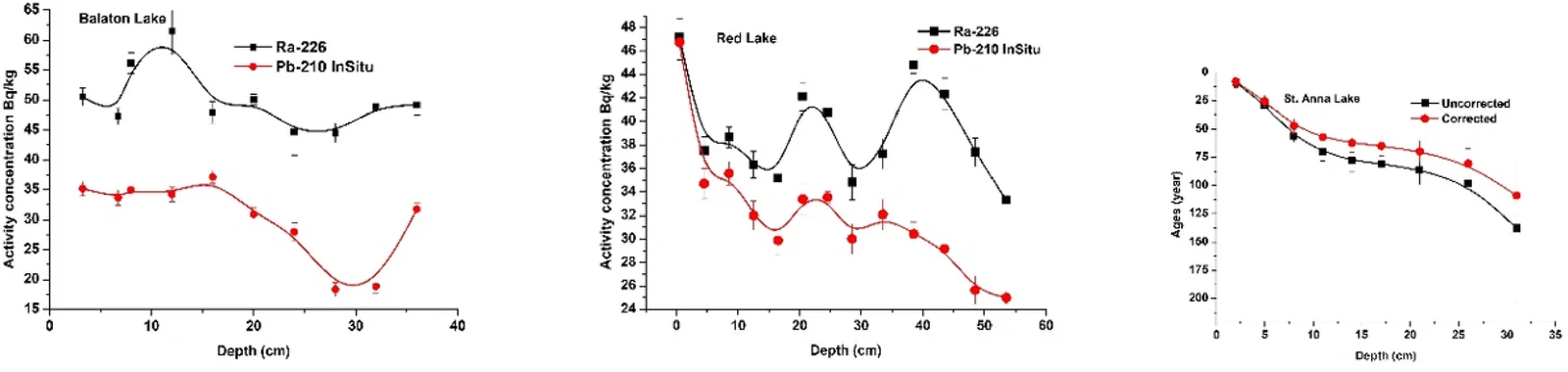
Activity concentrations of ^226^Ra and corrected in situ ^210^Pb.

By applying this factor and using the time parameter (*t*) derived from the ^137^Cs-based chronology, the corrected in situ ^210^Pb concentration can be determined. In Fig. 3, the calculated in situ ^210^Pb values are shown together with the measured ^226^Ra activities for each lake. The figures clearly demonstrate the significant discrepancy between these activities, and the substantial deviation that would result if ^226^Ra activity was directly assumed to be equivalent to in situ ^210^Pb. In the upper layers, the initial equilibrium between ^226^Ra and ^210^Pb within the grains is preserved. However, as we move downward through the strata, the ^210^Pb content of the grains decreases due to radioactive decay, and the small amount of upwelling ^222^Rn radon is unable to compensate for this decline. Consequently, the oldest layers are the most significantly affected by this phenomenon. In the case of Lake Balaton, the percentage differences between ^226^Ra and the actual in situ ^210^Pb range between 14% and 56%. For Lake St. Anna, these values vary from a minimum of 8% to a maximum of 87%, while for Red Lake the differences range between 1% and 32%. The largest deviations were observed in the case of Lake St. Anna, which can be explained by its low sedimentation rate and the high porosity of the sediments within the investigated interval. The smallest differences were detected in Red Lake, where the sedimentation rate is relatively high and the sediment exhibits a sandy structure. Lake Balaton represents an intermediate case among the previously mentioned lakes. Here, the sedimentation rate is relatively low; however, the sediment grain size and physical properties are closer to those observed in Red Lake. As mentioned above, the sediment is mainly composed of a mixture of precipitated calcium minerals. Such a discrepancy represents a substantial source of error, which, combined with other uncertainties, considerably increases the total error of the age model and undermines its reliability.

**Fig. 4:**
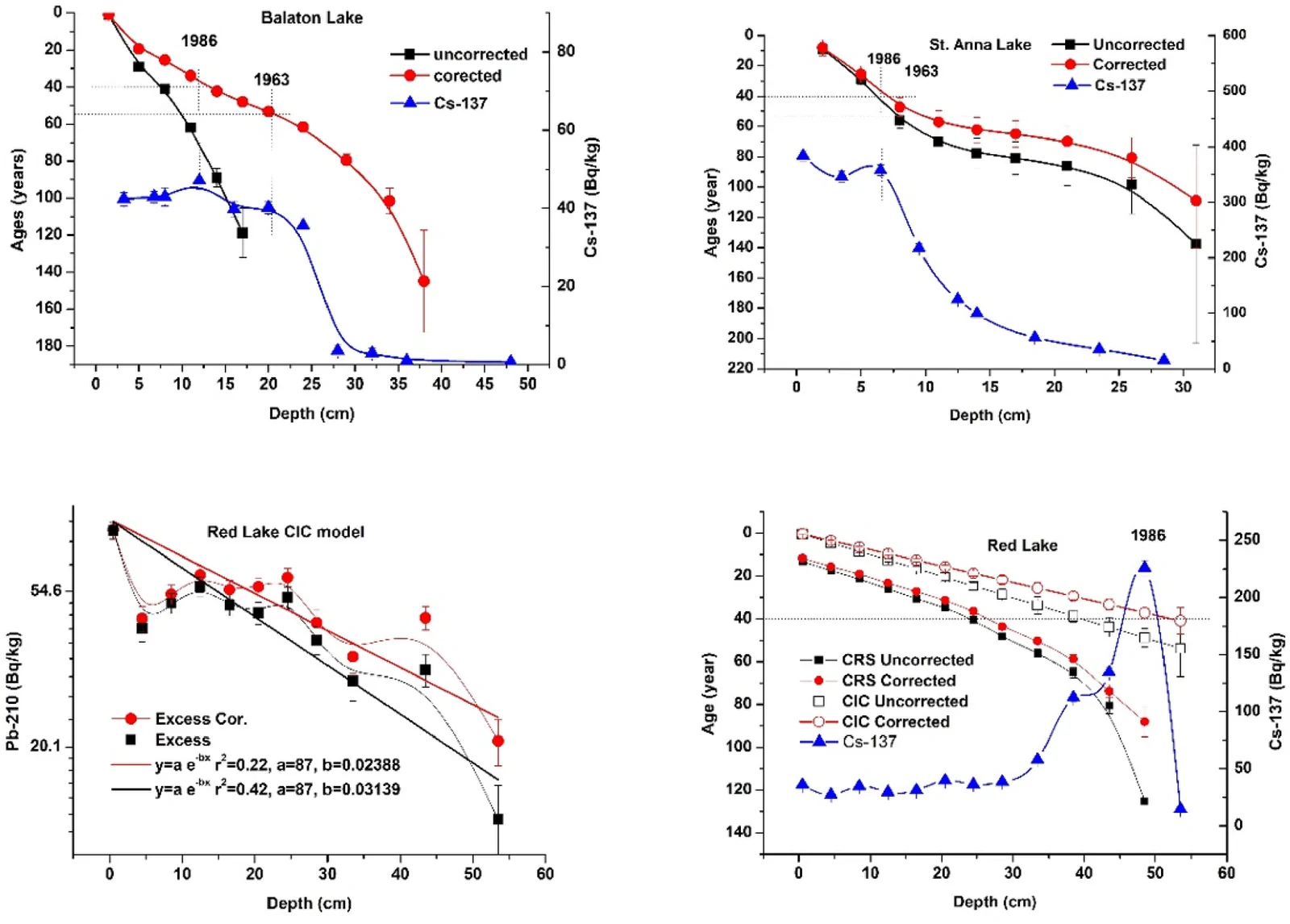
Age–depth models constructed using the CRS and CIC model for the investigated sediment cores.

The in situ ²¹⁰Pb deficiency resulting from the openness of the sediment system to ²²²Rn gas, and its consequences for the age model, are illustrated in Fig. 4, also incorporating the age values obtained from the ¹³⁷Cs peaks. Calculations were also performed under the assumption that the in situ ^210^Pb concentration is equal to the measured ^226^Ra activity concentration; this scenario is represented by the curve connecting the black data points in the figure. In this case, sediment ages are overestimated, resulting in an apparent acceleration of aging.

The Constant Rate of Supply (CRS) model is inherently prone to increased uncertainty near the dating horizon—that is, in the deepest layers datable by this method—because the atmospheric ^210^Pb inventory becomes small relative to the total inventory of the core. This explains the large uncertainties typically observed in the ages of the oldest datable layers. If radon loss is not accounted for, this effect is further amplified, ultimately compromising the reliability of the derived chronology.

The curve connecting the red data points in the figure represents the age model calculated using the corrected values. Marked discrepancies are observed across nearly the entire dated interval, with the most substantial deviations occurring in the deeper sediment layers.

Establishing the chronology poses a challenge in the case of Red Lake, as the complete sediment core spanning the last 200 years is unavailable. As mentioned above, the dating horizon was not reached. Consequently, the application of the CRS model is not feasible. In order to determine the age of the sediment layers, the Constant Initial Concentration CIC model was applied. This model allows for a closer approximation of the actual sediment age, which is further supported by the position of the 1986 ¹³⁷Cs peak within the sediment core.

According to the CIC model, the atmospheric ²¹⁰Pb concentration along the sediment core must be approximated by an exponential curve, where parameter *b* serves as the basis for the age model. Figure 4 displays the parameters of the CIC model applied to Red Lake; due to the variable sedimentation rate, the *r*^*2*^ coefficient of the approximation is not very high, though a better fit is obtained here as well using the corrected data. In this case, the model based on the corrected in situ ²¹⁰Pb yields the best agreement with the ¹³⁷Cs results, in contrast to the uncorrected model.

For Lake Balaton, our findings are corroborated by independent investigations based on other proxies^23^. In this case, obvious discrepancies arise between the two age models, and the errors of the uncorrected model are highlighted by the other proxies. In fact, this specific core is what initiated the present study. In this instance, the deviation between the correct and incorrect models reaches an error of 200%.

Based on the articles examining the distribution of radon gas in the sediment column^17,18,24^, this can be modeled by an advection-diffusion equation that takes advection and diffusion into account and also includes a decay term. In differential form, the equation can be written as follows:

$$\:{D}_{e\:}\frac{{d}^{2}C}{{dz}^{2}}-v\frac{dC}{dz}-\lambda\:C+S=0$$

Where *De* is the effective diffusion coefficient, *v* is the upward advection velocity of the water, *λ* is the decay constant of radon, and *S* is the radon production rate from the rock matrix. The solution to this equation can be written in the following form:

$$\:C\left(z\right)={C}_{\infty\:}(1-{e}^{-rz})$$

Where C_∞_ is the saturation concentration, which is the concentration where the production rate and decay reach equilibrium; that is, the theoretical equilibrium between ^226^Ra and ^222^Rn is established. In the equation, **r** represents the reciprocal diffusion length, which—neglecting the vertical upward flow of water—is defined as the square root of the ratio of the decay constant to the diffusion coefficient. The aforementioned function can be used to describe the radon concentration profile of the column and to provide an appropriate mathematical approximation. Using the ^226^Ra concentration, bulk density, and porosity, the saturation concentration can be written in the following form:

$$\:{C}_{\infty\:}=\frac{{A}_{{226}_{Ra}} \cdot \:{\rho\:}_{b} \cdot \:\epsilon\:}{n},\:r=\sqrt{\frac{\lambda\:}{{D}_{e}}}$$

Using the emanation factor ε from the formula, which was determined based on sediment grain size from literature data^25,26^, the theoretical radon concentration distribution within the sediment column can be established. By applying this to the pore water values measured in the Lake Balaton sediment, we obtained the approximation shown in Fig. 2 (C_∞_=30.27 Bq/l with 40 Bq/kg average ^226^Ra concentration 1.4 g/cm^3^ bulk density and 0.73 porosity, and *r* = 0.098 1/cm with D_e_=2.18 × 10^− 8^ m^2^/s. By choosing this value for a very loose, high water-content sediment: although diffusion in pure water is slower (10^− 9^ m^2^/s), the 73% porosity (which is almost a ‘dilute slurry’) and the less tortuous paths between the pores can justify this order of magnitude.). This approximation sufficiently reflects the measured values. Thus, if the values of the above parameters are known, a theoretical curve can be determined for the pore water radon concentration, which can subsequently be used to calculate the in situ ^210^Pb concentration.

## Materials and methods

### Study sites

Three lakes of contrasting origins were selected for this investigation, each exhibiting unique sediment sources and depositional environments. These variations in sediment provenance and formation processes provide a robust basis for a comparative analysis of sedimentation dynamics.

### Balaton lake

Lake Balaton, located in western Hungary, is the largest lake in both Hungary and the Pannonian Basin, and one of the largest shallow lakes in Europe. It has a surface area of approximately 589 km², a length of 78 km, an average width of 7.7 km, and lies 104 m above sea level^27^. The mean depth is 3.7 m at a water level of + 1.20 m. The lake’s catchment covers 5765 km² and exhibits an asymmetric morphology, being wider in the western region. The principal inflow is the River Zala, which drains nearly half of the entire catchment into the smallest basin, the Keszthely Basin^28^. The only outflow connects the Siófok Basin to the River Danube via the Sió Channel. Regulation of the maximum water level is achieved by the Siófok sluice; due to the steep artificial shoreline and basin morphology, water level fluctuations cause minimal change in surface area^27^.

The lake’s elongated basin structure and subdivision into distinct sub-basins strongly influence hydrodynamics, nutrient distribution, and the effects of water level variation. As a typical shallow lake, Balaton experiences frequent water column mixing and contains high concentrations of suspended material. A large portion of phosphorus is bound to these particulates, thereby reducing its bioavailability to phytoplankton^29,30^. According to the ²¹⁰Pb age–depth model, the average sediment accumulation rate between 1943 and 2017 was 5.8 years/cm, corresponding to 1.72 mm/year. Between 1900 and 1943, the rate was 2.5 years/cm (4 mm/year), and between 1866 and 1900, it was 11.5 years/cm (0.87 mm/year). These values were determined near the sampling point located at 46°53’40.3"N, 17°50’45.6"E^23^.

### Saint Anna lake

Lake Saint Anna (sampling point 46°07’37.9"N, 25°53’17.4"E) occupies the youngest volcanic crater of the Ciomadul Massif, part of the inner volcanic arc of the Eastern Carpathians. The lake formed as a crater lake fed exclusively by precipitation and surface runoff^31^. It developed within an extinct volcanic crater that was naturally filled with water following the last eruptive phase^32^.

The most recent volcanic activity of Ciomadul occurred between 10,000 and 35,000 (–42,000) years BP, as indicated by radiocarbon-dated charcoal and paleosol findings^32,33^. The bedrock consists of sandstone and conglomerate, overlain by amphibole–biotite dacite lava domes and pyroclastic deposits^34^. The crater is well-preserved, located at 950 m a.s.l., with a surface area of 0.193 km² and a catchment of 2.15 km². Lacking any natural outlet, the lake’s hydrological balance is maintained solely by precipitation and slope runoff^31^.

The sedimentary record extends back approximately 9300 calibrated years, displaying variable sedimentation rates throughout the Holocene^35^. These fluctuations are linked to hydrological shifts and climatic variability, as supported by multiple biological proxy indicators^36^.

### Red lake

Lake Red (sampling point 46°47’12.2"N, 25°47’11.4"E) is a natural dammed lake located in the Eastern Carpathians, northeastern Romania, at 983 m a.s.l. The lake was formed in 1837 when two landslides, triggered by a severe storm, blocked the course of the Bicaz River. Morphologically, the lake consists of two main basins: a southern basin approximately 1000 m long and a western basin about 442 m in length. The maximum depth is 9.6 m, the mean depth 5.41 m, the surface area 116,500 m², and the total volume 606,500 m³.

Sediment accumulation within the lake can be divided into two distinct phases. During the earlier period of relatively stable sedimentation (up to approximately 18 years ago), the accumulation rate ranged from 0.42 ± 0.1 to 0.55 ± 0.13 cm/year, averaging 0.48 ± 0.11 cm/year. In recent years, this rate has increased substantially, ranging from 1.2 ± 0.3 to 2.77 ± 0.6 cm/year, with an average of 1.68 ± 0.43 cm/year^37^.

### Sediment sampling and pore water ^222^Rn determination

Lake sediment sampling was conducted using a Uwitec USC 06000 gravity corer, with the retrieved cores being sectioned at 1-cm intervals. Special attention was given to ensuring that sufficient material was available for the required analytical procedures, including pore water radon concentration measurement, porosity determination, and radionuclide activity analysis.

To achieve this, a precisely weighed subsample of the homogenized sediment was first transferred into a low-potassium glass vial suitable for LSC (Liquid Scintillation Counting) measurements, taking care not to coat the inner wall of the vial with sediment above half of its height. This precaution is essential because porewater analyses were carried out using the “slurry” method combined with the LSC technique^16^.

The principle of the method is that the sediment sample placed in the vial is topped up with ProScint Rn (Triskem International) scintillation cocktail to the required volume (10 ml), followed by the addition of radon-free distilled water (previously boiled and cooled) while maintaining a maximum combined sediment and water volume of 10 mL. The prepared samples were measured after three hours using a TriCarb 2300TR liquid scintillation counter. The instrument was previously calibrated with a water sample of known radon activity.

Subsequently, the remaining sediment was used to determine water content and to calculate porosity. Accurate water content determination is essential, as the measured pore water radon activity must be corrected to account for the actual water volume present in the weighed subsample used for the analysis.

The dried samples were placed into sample holders of 11 mm diameter and 55 mm height, which were hermetically sealed with paraffin to prevent the escape of ^222^Rn gas from the samples. After a 30-day storage period to achieve secular equilibrium, the samples underwent gamma spectrometry.

## Calculation of insitu ^210^Pb

The activity of ^226^Ra, A_Ra_=A_0_, remains constant, where starting from $\:{t}_{0}$, a fraction * k*($\:0\le\:k\le\:1$) of the produced ^222^Rn is lost, and therefore does not contribute to the formation of in situ ^210^Pb.

Let:

- $\:{A}_{0}={A}_{\mathrm{R}\mathrm{a}}$- a ^226^Ra- activity (Bq).
- * k*-^222^Rn-escape fraction (*0 ≤ k ≤ 1*).
- $\:{\lambda\:}_{Pb}=\mathrm{l}\mathrm{n}2/{T}_{1/2}^{Pb}$- decay constant of ^210^Pb- ( $\:{T}_{1/2}^{Pb}\approx\:22.3$ year).
- $\:{N}_{Pb}\left(t\right)$- the number of ^210^Pb atoms at time t, with an activity of $\:{A}_{Pb}\left(t\right)={\lambda\:}_{Pb}{N}_{Pb}\left(t\right)$.

The initial condition assumes that at $\:{t}_{0\:}$radioactive equilibrium exists, thus $\:{A}_{\mathrm{Pb}}\left(0\right)={A}_{0}\Rightarrow\:{N}_{\mathrm{Pb}}\left(0\right)={A}_{0}/{\lambda\:}_{\mathrm{Pb}}$. Starting from $\:{t}_{0\:}$, due to ^222^Rn loss, the rate of ^210^Pb formation in the system is:

$$\:P=(1-k){A}_{0}$$

and the time evolution of ^210^Pb follows the usual differential equation:

$$\:\frac{d{N}_{Pb}}{dt}=(1-k){A}_{0}-{\lambda\:}_{Pb}{N}_{Pb}.$$

Solution for $$\:{N}_{\mathrm{Pb}}\left(0\right)={A}_{0}/{\lambda\:}_{\mathrm{Pb}}$$:

$$\:{N}_{Pb}\left(t\right)=\frac{\left(1-k\right){A}_{0}}{{\lambda\:}_{Pb}}+\left(\frac{{A}_{0}}{{\lambda\:}_{Pb}}-\frac{\left(1-k\right){A}_{0}}{{\lambda\:}_{Pb}}\right){e}^{-{\lambda\:}_{Pb}t}=\frac{{A}_{0}}{{\lambda\:}_{Pb}}\left[\left(1-k\right)+k{\hspace{0.17em}}{e}^{-{\lambda\:}_{Pb}t}\right].$$

Thus, the activity is:

$$\:{A}_{Pb}\left(t\right)={\lambda\:}_{Pb}{N}_{Pb}\left(t\right)={A}_{0}\left[\left(1-k\right)+k{\hspace{0.17em}}{e}^{-{\lambda\:}_{Pb}t}\right]{\hspace{0.17em}}.$$

$$\:{A}_{0}\equiv\:{A}_{\mathrm{R}\mathrm{a}}$$

$$\:{A}_{Pb}\left(t\right)={A}_{\mathrm{R}\mathrm{a}}\left[\right(1-k)+k{\hspace{0.17em}}{e}^{-{\lambda\:}_{Pb}t}].$$

- If $\:k=0\:$(no Rn loss): $\:{A}_{\mathrm{Pb}}\left(t\right)={A}_{0}$everywhere - no change.
- If $\:k=1\:$(all Rn lost):$\:{A}_{\mathrm{Pb}}\left(t\right)={A}_{0}{e}^{-{\lambda\:}_{\mathrm{Pb}}t}$- only decay remains, no replenishment.
- If radon loss is not a “constant fraction at production” but, for example, involves a continuous partial extraction (e.g. constant gas flow removing Rn), then the production term becomes time- or activity-dependent — in that case, replace $\:(1-k){A}_{0}$by the appropriate $\:P\left(t\right)$, and solve the differential equation accordingly.
- If the ^226^Ra activity is not constant (e.g. it is being removed or decreases significantly over the studied period), then $\:{A}_{0}$must be replaced by $\:{A}_{\mathrm{Ra}}\left(t\right)$, and the (now inhomogeneous) equation must be solved again.

### ^210^Pb, ^226^Ra, ^137^Cs analyses and ^210^Pb chronology

The isotopes necessary for age determination were measured using the aforementioned gamma spectrometric method. Following one month of storage—necessary to allow secular equilibrium to be established between ^226^Ra, ^222^Rn, and their decay products—the samples were measured using a high-resolution ORTEC GWL-120-15 well-type HPGe detector^5^.

During isotopic analysis, the following gamma peaks were monitored: 46.5 keV (^210^Pb), 661 keV (^137^Cs), and 295 keV and 351 keV (^214^Pb, for the determination of ^226^Ra). Activity concentrations were calculated via the relative method, utilizing the IAEA-312, 447, 375, and 385 Vienna reference materials. To achieve the required analytical precision, the counting time exceeded 120,000 s. To account for variations in core composition, matrix self-absorption corrections for ²¹⁰Pb at 46.5 keV were performed using matrix-matched certified reference materials or individual Monte Carlo simulations via GES-MC and Geant4 software.

Based on the measured data, the chronology of the samples was established using two independent methods. Special attention was given to the information provided by artificial isotopes. In our case, solely using ^137^Cs, a less detailed chronology can be established for approximately the past four decades. The introduction of ^137^Cs into the environment typically results in two activity maxima in lacustrine ecosystems. The first and more prominent peak, characteristic primarily of the Northern Hemisphere, is associated with the 1986 Chernobyl nuclear power plant accident. The deeper peak is linked to atmospheric nuclear weapons testing, which largely ceased following the 1963 Partial Test Ban Treaty.

Because two points define only a straight line, this approach yields a simplified age model. However, the resulting model allows for the determination of the time parameter (t) in the equation describing in situ ^210^Pb. The accuracy of this value minimally influences the calculated in situ ^210^Pb concentration, as the loss is most pronounced in the upper sediment layers, which typically represent the youngest deposits.

After applying the correction to the in situ ^210^Pb component, the age of the sediment layers was determined using the Constant Rate of Supply (CRS) and Constant Initial Concentration (CIC) models. The assumptions of the CRS model accurately reflect the processes occurring within the investigated catchment basins. The model assumes a constant annual atmospheric flux of ^210^Pb and accommodates variable sedimentation rates. For the studied lakes, both conditions are met.

## Conclusions

In this study, we demonstrated that enhancing the reliability and accuracy of chronologies derived from ^210^Pb dating is of paramount importance for the scientific community. While analytical measurement procedures have advanced significantly, we have shown that uncertainties arising from model assumptions can only be mitigated by selecting approaches that accurately reflect environmental conditions. Regardless of the specific model applied, the precise determination of the atmospheric ^210^Pb inventory remains a critical factor.

We identified and provided a comprehensive solution for a critical issue: the openness of sediment systems to ^222^Rn gas. By investigating three sediment cores with distinct genesis, we proved that this phenomenon is pervasive, though its impact varies depending on specific sediment parameters. Our results showed that corrected age models differ substantially from uncorrected ones, with deviations reaching up to 200% in the case of Lake Balaton. These corrected results were further validated by independent, proxy-based investigations, confirming the necessity of our proposed approach.

Furthermore, we introduced a correction procedure to mitigate inaccuracies in in-situ ^210^Pb concentrations caused by radon loss. Recognizing that pore water radon measurement is not a routine task, we developed a mathematical approximation based on easily measurable physical sediment parameters. This allows for a reliable estimation of the expected radon concentration and, consequently, the in-situ ^210^Pb content.

The implementation of this correction enables the construction of more accurate age models, particularly for older sediment layers. This study elevates the ^210^Pb dating method from a simple estimation tool into a highly precise and robust chronological approach, providing a more reliable foundation for paleoenvironmental reconstructions.

## Supplementary Information

Below is the link to the electronic supplementary material.

Supplementary Material 1

Supplementary Material 2

Supplementary Material 3

Supplementary Material 4

Supplementary Material 5

Supplementary Material 6

Supplementary Material 7

Supplementary Material 8

Supplementary Material 9

Supplementary Material 10

Supplementary Material 11

Supplementary Material 12

Supplementary Material 13

Supplementary Material 14

## Data Availability

All data supporting the findings of this study are available within the paper.
